# Analysis of Extreme Maternal Morbidity at the Women´s Hospital of Aguascalientes

**DOI:** 10.7759/cureus.16145

**Published:** 2021-07-03

**Authors:** Mayela G Cuesta-Galindo, Daniel E Bravo-Aguirre, Francisco J Serna-Vela, Omar O Camarillo-Contreras, José de Jesús O Yañez-Torres, María del Consuelo Robles-Martínez, Alejandro Rosas-Cabral

**Affiliations:** 1 Gynecology and Obstetrics Department, Women's Hospital of Aguascalientes, Aguascalientes, MEX; 2 Investigation Department, Health Services Institute of the State of Aguascalientes, Aguascalientes, MEX; 3 Gynecology and Obstetrics Department, Women´s Hospital of Aguascalientes, Aguascalientes, MEX; 4 Medicine Department, Autonomous University of Aguascalientes, Aguascalientes, MEX

**Keywords:** maternal near miss, extreme maternal morbidity, maternal mortality, obstetrics, hypertensive disorders

## Abstract

Background

Extreme maternal morbidity is defined as "events that potentially threaten the life of a pregnant woman during pregnancy, childbirth or the puerperium, but that due to a medical intervention the patient does not die", and this is an indicator of health quality at the hospital and demographic level.

Objective

The aim of this study was to determine the prevalence of extreme maternal morbidity in the Women´s Hospital of Aguascalientes, Mexico.

Material and methods

A retrospective cross-sectional study was conducted under the criteria of the World Health Organization and the Latin American Federation of Obstetrics and Gynecology Societies for the definition of extreme maternal morbidity to determine the prevalence of near miss morbidity, between January 1 and December 31, 2016.

Results

We found 165 cases of extreme maternal morbidity; no maternal death was registered during the study year. The extreme maternal morbidity rate was 0.016 and 16.69 per 1000 live births; the ratio of extreme maternal morbidity cases / obstetric admissions was 11.07. The prevalence of extreme maternal morbidity was 1.6%. The main causes of extreme maternal morbidity were hypertensive disorders (57%), obstetric hemorrhage (29%), sepsis (1%) and other (13%).

Conclusion

Extreme maternal morbidity in our institution had a similar prevalence to that reported in other countries and was mainly caused by hypertensive disorders.

## Introduction

Maternal Mortality is an indicator of the level of economic development of a given country, of the reach of its health infrastructure and the level of inequality of such [1].

In order to learn about the behavior of maternal death, different approaches have been developed, such as “verbal autopsy” or extreme maternal morbidity (near miss) [2]. The World Health Organization (WHO) defines extreme maternal morbidity as an event that potentially threatens the life of a pregnant woman, be it during the pregnancy, childbirth or within the following 42 days after the end of the pregnancy itself, but, thanks to a medical intervention, the woman does not die [3].

Extreme maternal morbidity rate is also related to the level of development of a given region. In 2014, maternal morbidity rates of 4.9% were reported in Latin America, 5.1% in Asia, 14.9% in Africa, 1.4% in North America and 0.8% in Europe [4]. Depending on the context and criteria used, the incidence of extreme maternal morbidity varies between 0.6% and over 30% [5].

The progression of normality to maternal morbidity is related to the type of event, social factors, demographic circumstances (regions of difficult access for treatment), characteristics of the service provider (tardy diagnosis of morbidity and/or delay regarding the initiating of treatment), supplies available in health institutions and the patient´s attitude towards the system (inadequate prenatal control, not recognizing alarm data and/or not requesting timely medical attention) [6]. Approximately 50 million maternal health problems are reported around the world every year and almost 300 million women suffer, in short and long term, of illnesses and lesions related to pregnancy, childbirth and puerperium [7]

Amongst the Millennium Development Objectives, as a world effort, is that to improve maternal health as a part of a global Alliance for development, it being one of the most urgent human needs and one of the fundamental rights of all human beings. [8]

The study of extreme maternal morbidity may be an indicator to complete the analysis of maternal deaths with the intention of evaluating health services and the reach of sanitary and assistance infrastructure [9]. Knowing the physiopathology of each condition, the evaluation of the patients' characteristics, medical attention they received and the follow-up of conditions of those who survived will allow that information to be used to determine the factors contributing to maternal morbidity, thus helping to establish prompt prophylactic and strategic measures that will allow the decrease in complications and sequels in short and long terms, achieving a real impact in avoiding new cases of maternal deaths, at local, regional and national levels. Thus, the objective of this study is to determine the prevalence of extreme maternal morbidity at the Women´s Hospital of Aguascalientes in the period from January 1st to December 31, 2016.

## Materials and methods

A retrospective cross-sectional study was conducted, where files from the obstetric intensive care unit of the Women´s Hospital of Aguascalientes were reviewed in order to identify women who met the criteria of Extreme Maternal Morbidity established by the World Health Organization (WHO), and the Latin American Federation of Obstetrics and Gynecology Societies (FLASOG), between January 1st and December 31, 2016. Sampling was not conducted since it was possible to study the total population.

Population was divided in three categories (specific illness, organ/system failure or malfunction and established management) according to the presence of one or more criteria from the WHO/FLASOG (Table 1). Some of the patients complied with the characteristics to be included in more than one category, thus, having more than one variable of each category. 

**Table 1:** Criteria to identify cases of Extreme Maternal Morbidity according to WHO /FLASOG classification.

Criteria related to the specific illness	
Pre-eclampsia with severity data	Systolic blood pressure ≥160 mmHg or diastolic blood pressure ≥ 110 mm Hg, platelets <100,000. Duplicate liver enzymes, persistent epigastralgia with no explanation, creatinine >1.1 mg/dL or duplication in absence of renal illness, pulmonary edema or neurological or visual alterations
Eclampsia	Generalized convulsions, coma or both in absence of other neurological disorder
Hypovolemic shock	Secondary syndrome to acute loss of circulating volume. Clinical data: arterial hypotension, tachycardia, oliguria, deterioration in the state of consciousness, paleness, distal hypothermia and delayed capillary refilling
Septic shock	Sepsis induced hypotension, despite adequate intravenous liquid support (systolic blood pressure <90 mm Hg, diastolic blood pressure <60 mm Hg), fever (38°C), hypothermia (<36°C), tachypnea (>20), tachycardia (>90) leukocytes >12,000 or <4,000 or >10% of bands
Criteria related to organ/system failure or malfunction	
Heart	Heart arrest, pulmonary edema with intravenous diuretic use, inotropic, vasopressor o vasodilator support.
Vascular	Absent peripheral pulse or hypotension for 30 minutes associated to hypovolemic or septic shock, median arterial pressure <60 mm Hg, capillary refilling >2 seconds, need of vasoactive support. Also consider where blood arterial pressure >160 mm Hg or diastolic arterial pressure >110 mm Hg persisting for more 20 minutes.
Renal	Acute deterioration of renal function, 50% creatinine increase in 24 hours or elevation >1.2 mg/dL, oliguria (< 0.5 ml/kg/hour) not responding to intravenous liquid replacement and intravenous diuretics, acid-base or electrolytic disorder
Liver	Skin and scleral jaundice, or total bilirubin > 3 mg/dL, transaminase > 70 UI/dL, lactic dehydrogenase > 600 UI/L
Metabolical	Joint comorbidities such as diabetic ketoacidosis, thyroid crisis, that may manifest as part of alterations of the underlying illness, lactic hyperlactacidemia > 200 mmol/L, or hyperglycemia > 240 mg/dL not having diabetes
Cerebral	Coma, convulsions, confusion, disorientation in person, space or time, focalization signs, hemorrhagic or ischemic lesions.
Respiratory	Respiratory failure syndrome, need of invasive or non-invasive ventilatory support, respiratory arrest
Coagulation	Disseminate intravascular coagulation, (prolonged coagulation time , fibrinogen decreased, D-Dimer elevation) platelets <100,000 /mm3, Lactic dehydrogenase > 600 UI/L
Criteria Related to established treatment	
Admittance to obstetric intensive unit care	Patient who enters due to the need to administer amines, or mechanic ventilation, intubation and advance cardiopulmonary resuscitation, do not count as criteria for patient who enter to elective hemodynamic stabilization
Surgery	Procedures practiced with urgency for management of obstetrics complications or for any condition which generates a serious compromise of the pregnant woman; procedures different to curettage, delivery or cesarean.
Transfusion	Transfusion of 3 or more units of any blood component facing an acute event

The study was approved by the Ethical Research Committee of the State of Aguascalientes, obtaining a registry based on the criteria for the identification of extreme maternal morbidity cases. According to criteria from WHO/FLASOG, variables were added to obtain information on characteristics regarding socio-demographic and obstetric histories of each of the participants in the study.

For the statistical analysis, data were entered and analyzed using the SPSS program version 21.0 (IBM Corp. Armonk, NY), frequencies and percentages were calculated for categorical variables, Chi square was used for the comparison of proportions and a *p*-value of <0.05 was considered statistically significant; mean and standard deviation was calculated for continuous variables. The outcome indicators were calculated using the formulas:

Extreme maternal morbidity ratio = Number of extreme maternal morbidity cases /total number of live births x 1000

Ratio of cases of extreme maternal morbidity/ obstetric admissions = Total cases of extreme maternal morbidity/ number of obstetric admissions x 1000. 

Ratio of maternal deaths per 1000 live births = Number of maternal deaths cases / total number of live births x 1000

## Results

During the study period, a total of 14,901 pregnant women were attended in our hospital, and 9,882 births were recorded, of which, 6,331 were eutocic deliveries (64.0%), 109 were dystocic deliveries (1.1%), 3,442 (34.9%) were cesarean sections and 953 were abortions.

One hundred sixty six cases of extreme maternal morbidity were identified, and 165 files were analyzed (an incomplete file was excluded); no maternal death was registered during the year of study.

The average age of patients was that of 25.13±6.56 with a range between 14 and 46 years of age; 85% of patients with extreme maternal morbidity were 34 years of age or younger. Other socio-demographic characteristics of patients may be observed in Table 2.

**Table 2:** Socio-Demographic characteristics of 165 cases of extreme maternal morbidity at the Women´s Hospital of Aguascalientes from January 1st to December 31st, 2016.

Characteristic	Number (%)
Age (years, average, standard deviation, range)	25.13±6.56 (14-46)
14-19	26% (42)
20-34	59% (98)
>35	15% (25)
Level of Studies	
Primary	19% (31)
Secondary	56% (93)
Technical Career	2% (4)
High school	17% (28)
Bachelor´s Degree	4% (7)
Postgraduate	1% (1)
Illiterate	1% (1)
Marital Status	
Cohabitation	55% (90)
Married	23% (39)
Single	21% (35)
Separated	1% (1)
Occupation	
Housewife	84% (138)
Employee	6% (10)
Student	5% (8)
Merchant	4% (7)
Professional	1% (2)

The number of pregnancies each woman had was between 1 and 11, with a median of 2 per patient; 40% were primiparous (66), 39% (64) with 3 or more pregnancies and 21% (35) on their second pregnancy (*X*^2^ =0.05, p=0.9)

Regarding prenatal control, the number of medical consultations varied between 0 and 19, with an average of 5 consultations, within that established in the Official Mexican Norm; 40% (66), initiated prenatal control previous to the 12 weeks into pregnancy and 60% (99) received medical prenatal consultation in a tardy fashion. According to the type of prenatal control, 59% (97) of the patients received optimal prenatal control (equal or higher to 5 consultations), 36% (60) sub-optimal (1-4 consultations) and 5% (8) denied having received any prenatal control attention (Table 3).

**Table 3:** Number of prenatal consultations in 165 cases of extreme maternal morbidity at the Women´s Hospital of Aguascalientes

Number of Consultations	N	%
≥ 5 consultations	97	59
1 -4 consultations	60	36
None consultations	8	5
Total	165	100

Gestational age at the end of pregnancy had a mean of 33 weeks, varying between 5 to 42 weeks. Of these, 44% (72) were resolved at maturity, 39% (64) were premature, 6% (10) at fetal immaturity, 7% (12) were not viable or within the first trimester and 4% (7) continued pregnancy after the event.

The indication to end of pregnancy was reported in the following order: hypertensive disorders 53% (87), spontaneous labor 18% (30), placental alterations 14% (23), iterative 13% (22), and other causes 2% (3).

As to the means of interruption of pregnancy, the most used was cesarean with 71% (117), natural deliveries 18% (30), laparotomy due to ectopic pregnancy 4% (7), uterine curettage evacuation 3% (4), and pregnancies not concluded at 4% (7). 72% (119) of the patients were healthy at the moment of the event and 28% (46) presented a previous co-morbidity.

Regarding the perinatal result, 78% (127) were live products, 6% (8) stillborn, followed by 4% (7) with ruptured ectopic pregnancy and 2% (4) abortions; also registered were 3% (6) of early neonatal deaths and another 3% (6) of late neonatal deaths. Of the total of the cases in the study, 4% (7) continued with the remaining pregnancy having received medical treatment and overcoming the extreme maternal morbidity scenario; no maternal deaths were registered.

The extreme maternal morbidity ratio was 16.69 per 1,000 live births, the relation of cases of extreme maternal morbidity/ obstetric admissions was that of 11.07, and the prevalence of extreme maternal morbidity was that of 1.6%. (Table 4)

**Table 4:** Extreme maternal morbidity indices among patients admitted in the Women’s Hospital of Aguascalientes from January 1st to December 31, 2016.

Parameter	N
Number of live births	9882
Number of maternal deaths	0
Number of maternal near-miss cases identified	165
Number of obstetric admissions	14,901
Extreme maternal morbidity ratio/ 1000 live births	16.69
Extreme maternal morbidity ratio/obstetric admissions	11.07
Prevalence of extreme maternal morbidity	1.6

Regarding the diagnosis, we looked for diseases related to pregnancy, delivery and puerperium linked to a direct or indirect cause of obstetric morbidity. Of these, 87% (144) were due to a directa cause and 13% (21) were related to an indirect cause. Direct causes were distributed as follows: hypertensive disorders 57% (94), obstetric hemorrhage 29% (48), obstetric sepsis 1% (2). Of the cases due to an indirect cause, 12% (19) correspond to other complicated illnesses in pregnancy (diabetic ketoacidosis, thyroid crisis, congenital heart disease, acute pulmonary edema, pulmonary hypertension, pancreatitis, epilepsy, among others) and 1% (2) included autoimmune/ hematological pathology (Table 5)

**Table 5:** Extreme maternal morbidity causes among 165 women admitted in the Women´s Hospital of Aguascalientes from January 1st to December 31, 2016

Cause	N (%)
Direct cause	
Hipertensive disorders	94 (57)
Obstetric hemorraghe	48 (29)
Sepsis	2 (1)
Indirect cause	21 (13)
Total	165 (100)

According to WHO/FLASOG criteria, 351 records were included in the 165 patients, of which, 146 (41.5%) corresponded to specific illness, 147 (41.8%) to organic failure or dysfunction and 58 (16.3%) to established management. According to specific illness, the main pathology registered was preeclampsia with severity criteria 58.8% (86), hypovolemic shock 32.8% (48), eclampsia 5% (7), HELLP (hemolysis, elevated liver enzymes, low platelet count) syndrome 0.7% (1), septic shock 1.4% (2), and 1.4% (2) an indirect cause.

Related to organic failure or dysfunction: 147 met criteria related with organ or system failure or dysfunction; 30 % (45) at vascular level, renal 19% (28), coagulation 15% (23), hepatic failure 12% (17), alterations at cerebral level 9% (13), metabolic dysfunction 7% (10), respiratory dysfunction 5% (7) and cardiac failure 3% (4).

Related to established management, 58 complied with criteria to classify depending on the established management: 62% (36) required blood transfusion, 26% (15) required additional surgery, and 12% (7) needed admission to the intensive care unit; three required intubation or assisted mechanical ventilation, three required administration of vasoactive amines and one required both procedures.

Of the patients studied, 66% (109) required other procedures to manage their severity and pathology, 52% (57) with anticonvulsant prophylaxis, 32% (35) continuous intravenous administration of antihypertensive, 12% (13) required uterotonics for active management of obstetric hemorrhage and for 4% (4) it was necessary to conduct Zea clamping. 

All the patients studied were sent to intensive care in an elective fashion for hemodynamic stabilization, with an average stay of 3 days, varying between 1 and 9 days. Total hospitalization stay was on average of 6 days, with a range of 1 to 15 days. 

## Discussion

According to estimates by the WHO, maternal complications during pregnancy, childbirth and puerperium appear even in 15% of all pregnancies [10]. Extreme obstetric morbidity is considered an instrument to evaluate the level in quality of medical attention. Documenting of extreme maternal morbidity using “near miss” criteria by the WHO has gained acceptance as a valuable tool for documenting the quality of attention given to pregnant women in a certain region or country since it allows for the understanding of factors that contribute to a fatal maternal outcome [11]. In this study, we found a prevalence of 16.69 cases per 1000 live births, which is a value found within the range of prevalence recently reported in a systematic revision published by Abdollahpuor et al. [12], who observed that the pondered prevalence of global extreme maternal morbidity was that of 18.67 cases per 1000 live births, with an Confidence Interval (CI) of 95% from 16.28 to 21.06. Nevertheless, it is superior to that reported in Brazil and South America (8.69/1000 live births and 11.57/1000 live births respectively), which are countries with a development level similar to that of our country. Likewise, this rate is way superior to that observed in Europe (3.10/1000 live births), but inferior to that observed in India (28.22/1000 live births) or Africa (31.88/1000 live births), which translates the need to reinforce strategies that have been implemented in our country in order to decrease extreme maternal morbidity [13].

75% of our patients with a “near miss” event reported a level of education of secondary school or less and only 24% of them refer a high school or university education, which could mean that, in our population, a low education level is a risk factor for a severe pregnancy complication or a high risk of maternal mortality, which coincides with that stated by Monroy et al. in our country [6], by Dessalegn et al. in Ethiopia [14], and by Benimana et al. in Rwanda [15].

A total of 71% of our population required delivery by abdominal delivery, which is related to the severity of the maternal morbidity and linked to the fact that cervix conditions of those patients are not favorable for inducing labor. It is nevertheless worth mentioning that some authors have stated that the fact of conducting a cesarean is a risk factor for complications to present themselves leading to “near miss” events, mostly in regions where an important increase in the rate of cesarean procedures has been observed [16].

In the review of the main causes of extreme maternal morbidity, we found hypertensive disorders in first place with 57% followed by obstetric hemorrhage with 29%, which coincides with that reported by other authors in Latin America [17] and Morocco [18], although it is worth mentioning that it is different to that reported by other authors who mention as a first cause of extreme maternal morbidity to be obstetric hemorrhage [19-22]. This coincides with the high incidence of pre-eclampsia in our country along with the fact that in our study, no maternal deaths were registered, and demonstrates that even though morbidity due to this pathology has not decreased, the established management in our hospital has had an impact on reducing the rate of maternal deaths and reinforces the need to maintain close surveillance of these patients (Table 6).

**Table 6:** Maternal mortality rates in the Women´s Hospital of Aguascalientes from 2011 to 2016

Parameter	2011	2012	2013	2014	2015	2016
Number of Live births	10291	10767	10741	10799	10063	9882
Number of Maternal deaths	2	7	1	1	1	0
Maternal mortality ratio per 1000 live births	0.19	0.65	0.09	0.09	0.09	

Using criteria established by the WHO, 89% was categorized as organ or system failure or dysfunction, 88% as specific illness and 35% was related to established management, which is different to that reported by Witteveen et al. who refer a frequency of 87.2% for specific illness criteria, 78.9% for those related to established management and 38.2% for criteria based on organ or system dysfunction in a study that compares these criteria in countries with high and low incomes [23]. This indicates that in our institution, “near miss” events are more frequent due to conditions pertaining to the patients rather than faults in treatment.

A total of 59% of our patients had an optimal prenatal control according to the Official Mexican Norm regarding medical attention for pregnant women (NOM-007-SSA2-2016)[24] , which coincides to that reported by Shen et al. who state that patients that had less than six medical consultations for prenatal control have a greater risk of presenting a “near miss” event (6.76 Confidence interval 95%,0.87 a 21.8) [25], which in turn coincides with the average number of prenatal consultations considered as an optimal prenatal control in our country (5 consultations). It is worth mentioning that the initiation of prenatal control of our patients was tardy, implying the risk for development of “near miss” events. Therefore, criteria must be revised regarding the consideration of optimal prenatal control in our country which only considers the number of control consultations during pregnancy and not the timeliness of initiation of such control. 

Another individual determinant for obstetric risk to consider is parity. Most authors suggest that multi-parity is a risk factor for extreme maternal morbidity [26-28]. Although, there are authors who state that first time pregnancy is also a risk factor associated with this condition [29], we found no significant difference between history of being multiparous and primiparous for the development of an extreme maternal morbidity event ( *X*^2^ =0.05, p= 0.9).

An important challenge to reduce maternal deaths is that of limited access to emergency obstetric services. Therefore, for adequate care of patients with extreme maternal morbidity, it is of utmost importance to have intensive and intermediate care units. Even though the number of ICUs has been increased, they are not adequately managed for critical obstetric conditions even in developed countries. There are studies that demonstrate that the use of ICUs in women with severe complications protects and reduces maternal mortality but this is not valid for women with less severe conditions. It is not enough to “provide intensive care to any woman with a complication, it is better to clearly judge the use of resources or well established criteria regarding the use of an ICU for obstetric patients at general hospitals to guarantee that the Unit is available for whom needs it the most” [30, 31]. The lack of obstetric ICUs may increase the maternal mortality rate because patients will be treated in non-adequate areas, without trained personnel to control the event, such as recovery wards, rooms for isolated patients, and sometimes, common areas due to lack of physical space in critical areas. It is necessary that all hospital units that attend to obstetric patients have specific physical, technological and human resources infrastructures for the attention of extreme maternal morbidity events. In our institution, all patients were taken to this area, from elective hemodynamic stabilization to the management of severe complications or pre-surgical stabilization, which helps to keep a low maternal mortality rate, even though the rate of extreme maternal morbidity events is still high with respect to rates of other institutions and regions of the world [12]. Availability and appropriate use of good quality ICUs are crucial for the reduction of maternal mortality.

The limitations of our study are it being retrospective and limited to only one center, the brief period of study and thus, a low number of patients included. Nevertheless, it represents the experience of the institution with the larger infrastructure and work load in this region of our country. 

## Conclusions

Extreme maternal morbidity still affects a high number of patients that seek medical attention at the Women´s Hospital of Aguascalientes. Hypertensive disorders are the main cause for their state of health to be altered and this is significantly related to the morbidity severity of obstetric patients in our country. These elevated morbidity rates, along with a low maternal mortality rate make it necessary to evaluate the availability and appropriate use of intensive care units in hospitals that provide attention for pregnant women in our state, region and country.
